# Mechanical Properties and Reaction Kinetics of Alkali-Activated Metakaolin

**DOI:** 10.3390/ma17020367

**Published:** 2024-01-11

**Authors:** Chao Cui, Yingze Dang, Chenguang Luo, Lan Wang, Hui Peng

**Affiliations:** 1 Key Laboratory of Civil Engineering Structure and Mechanics, Inner Mongolia University of Technology, Hohhot 010051, China; cui6201798@163.com; 2 School of Civil Engineering, Inner Mongolia University of Technology, Hohhot 010051, China; d221737@163.com (Y.D.); 20221800461@imut.edu.cn (C.L.); 3 Key Laboratory of Bridge Engineering Safety Control, Ministry of Education, Changsha University of Technology, Changsha 410114, China; huipeng@csust.edu.cn; 4 School of Civil Engineering, Changsha University of Technology, Changsha 410114, China

**Keywords:** metakaolin, geopolymer, mechanical properties, reaction kinetics

## Abstract

In this study, the influence of the physicochemical properties and proportioning conditions of metakaolin on the mechanical properties of the synthesized metakaolin geopolymers was comprehensively evaluated, and the issue of the reaction control mechanism for the formation of mechanical properties during the synthesis of geopolymers was addressed. The reaction mechanism was analyzed by SEM and FTIR, and the kinetic analysis of the geopolymerization process was carried out using isothermal calorimetry combined with the Jander model. The test results show that the physicochemical properties of the metakaolin and the proportioning conditions together affect the mechanical properties of the geopolymer, with the correlation between the active aluminum content of the metakaolin and the strength of the geopolymer reaching over 0.87. The early stages of the geopolymerization reaction are all controlled by nucleation–growth mechanisms (*N* < 1), and the variability in control mechanisms is mainly found in the later stages of the geopolymerization reaction. The low reactivity and slow exothermic hydration of metakaolin are more inclined to the nucleation-growth mechanism responsible for the hydration process due to the large amount of encapsulation.

## 1. Introduction

Ordinary Polenta cement (OPC) is one of the most frequently used materials in the construction industry. However, its production is energy-intensive, consuming approximately 12–15 percent of the total industrial energy [1] and 8 percent of global CO_2_ emissions [2]. Therefore, in recent years, the scientific community has been encouraging the production of low-cement or clinker-free cementitious materials. As a potential alternative to OPC, geopolymer is a low-carbon binder and clinker-free [3,4]. It is produced by exciting solid aluminosilicates such as metakaolin, fly ash, and granulated blast furnace slag with alkaline solutions of silicates, carbonates, and alkali metal hydroxides. Among them, metakaolin has more silica and alumina fractions, lower impurity content, a stable chemical composition, higher purity compared to fly ash and slag, and the reproducibility of synthetic geopolymers, which makes it one of the most frequently used precursors for synthetic geopolymers [5].

To promote the application of metakaolin-based polymers in practical engineering, it is necessary to establish a link between their macroscopic properties and microstructure and the preparation process and raw materials, to fundamentally reveal the influence mechanism of each factor on the metakaolin-based polymer. The mechanical properties of the geopolymer are closely related to its early hydration reaction. However, the issue of the early hydration kinetics behavior controlling the mechanical properties and microstructural development of metakaolin-based polymers is not well understood [6,7]. The exact mechanisms involved are also unclear.

In recent years, some progress has been made regarding the hydration kinetics of geological polymerization processes, and a range of hydration kinetic models have been applied. Examples of kinetic models are Jander [8], JMAK [9,10], Krstulovic–Dabic [11,12], and Ginstling–Brounshtein [13]. Where the Jander model is suitable for systems based on the conservation of mass, the modified Jander model is commonly used for the modeling of cementitious materials and can also be used for geopolymer synthesis processes in highly alkaline environments. Isothermal calorimetry, which allows the continuous determination of the extent of geopolymer reactions, has been used extensively in the literature to monitor the hydration process of geopolymers [14,15]. Unlike other conventional methods (e.g., selective dissolution, which requires the specification of silica–aluminum material fineness, and, secondly, acid dissolution, which is more difficult to control with precision), the availability of sufficient and continuous data is one of the conditions for the accurate estimation of the kinetics. Researchers have investigated the early hydration kinetics of geopolymers using isothermal calorimetry. For example, Ravikumar [16] used isothermal calorimetry to calculate the modified Jander’s equation and concluded that phase boundary reactions dominate the hydration process of geopolymers. From the dissolution stage of the geopolymerization reaction, phase boundary reactions control the hydration process of geopolymers. Nath [17,18] and others have argued that the reaction process in alkali-activated fly ash systems is controlled entirely by the nucleation–growth mechanism and does not involve a diffusion mechanism to control the hydration reaction. It has also been shown that the synthesis states of geopolymers at different stages of the process are different in terms of the mechanisms that kinetically control the progress of the reaction [19]. There are different views on the kinetic characterization of silica–aluminate systems, and the clarification of the mechanisms controlling the hydration reaction of metakaolin has become an important issue.

This study investigated the formation mechanism of the mechanical properties of metakaolin synthetic geopolymers, and the kinetic mechanism of the early hydration of metakaolin-based geopolymers is further explored. The effects of the proportioning conditions and the physicochemical properties of the materials on the mechanical properties of the geopolymers were comprehensively assessed by compressive strength tests, FTIR, and SEM; after this, isothermal calorimetry (ICC) was used to evaluate and quantitatively analyze the polymerization process dynamically. Because ICC can reliably quantify the degree of thermochemical reaction of alkali-activated metakaolin geopolymers, a Jander mathematical model was also applied to study the reaction mechanism and kinetics of the polymerization process.

## 2. Testing

### 2.1. Raw Materials

The silica–aluminate raw materials used in this work were sourced from Datong (Shanxi, China) and Imerys (Paris, France), with the two kaolin materials being Devolite (Imerys) and Shanxi (Shanxi) and three metakaolin materials being M501, 1200S, and Opacilite (Imerys). The chemical composition of the above raw materials was determined using X-ray fluorescence spectrometry (XRF), the results of which are shown in Table 1. A strong alkaline activator was prepared using a solution of water glass with a modulus of 2.8, industrial flake sodium hydroxide (98% purity), and deionized water.

### 2.2. Methods of Characterizing Raw Material Properties

**Figure 1:**
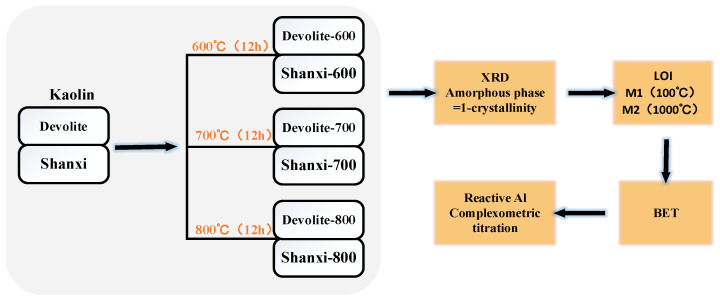
Calcined kaolin and physical and chemical property determination process.

In this study, two kaolin clays, Devolite and Shanxi, were calcined at 600 °C, 700 °C, and 800 °C for 12 h to obtain six metakaolin clays, as shown in Figure 1. Table 2 gives the physicochemical properties of the nine metakaolin clays used in this study. The data in the table indicate that the amorphous and soluble aluminum content of the same kaolin clay vary after calcination at different temperatures, which makes it easy to compare and analyze the influence of the chemical components (amorphous and soluble aluminum content, etc.) on the level of the geopolymerization reaction and the mechanical properties of the reaction products, provided that the physical properties (particle size and specific surface area, etc.) of the raw materials are the same.

**Figure 2:**
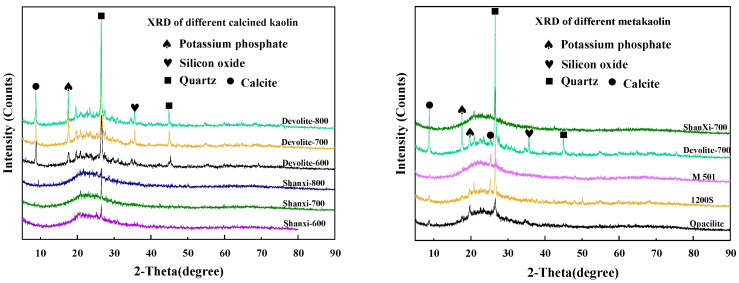
XRD graph of different metakaolin materials.

X-ray diffraction (XRD) characterization: the nine metakaolin materials mentioned above were characterized using a D/MAX2550VB+ diffractometer manufactured by Rigaku Corporation, Japan, with a scan range of 2theta = 5° to 95°, and the results are shown in Figure 2. As shown in Figure 2, the quartz phase was found at 2theta = 26.5° for both calcined kaolin and metakaolin. The kaolin raw material mainly introduces the quartz phase and does not affect the polymerization reaction. Multiple diffraction peaks are present in the Devolite-type kaolin. The calcite phase at 2theta = 9° was analyzed. In addition, a Si-bearing phase at 2theta = 35° and a potassium phosphate phase at 2theta = 17.5° were found in the Devolite-type kaolin. This also indicates that the composition of Devolite-type kaolin is more complex than that of other kaolins. The Jade 6.5 software was used to analyze the mineral composition and calculate the mean particle size (D) and crystallinity. Amorphous phase content = 1 − crystallinity. Loss on ignition (LOI) analysis: the metakaolin was dried at 100 °C and weighed as mass M_1_; it was then placed in a muffle furnace and scorched at 1000 °C and weighed as mass M_2_, LOI = M_1_ − M_2_. Specific surface area (BET) characterization: the Quadrasorb SI-3MP analyzer from Conta, Gainesville, GA, USA, was used, and the results are shown in Table 2. Reactive aluminum content analysis: determination by the “titration complex method” [20].

### 2.3. Geopolymer Preparation Method

**Figure 3:**
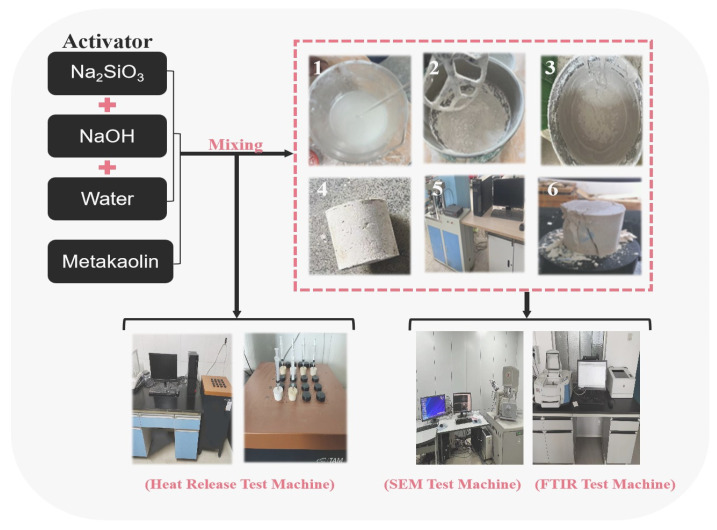
Preparation of geopolymers and determination of compressive strength, SEM, FTIR, and exothermic hydration.

A water glass solution with a modulus of 2.8 was used. NaOH was added to adjust the modulus (M = 1.2, 1.4, 1.6, and 1.8), which was later mixed with deionized water to prepare alkaline activators with different concentrations (C = 50%, 60%, 70%, and 80%), as shown in Figure 3. The mixing of metakaolin with the alkaline activator was performed at a liquid–solid ratio of 0.5 (mL:g). They were mixed well in the slurry mixer; then, we slowly injected the slurry into a φ50 mm × 50 mm cylindrical mold. The ground polymer matrix was compacted by vibration for 120 s. It was placed at different curing temperatures (T = 20 °C, 40 °C, 60 °C, 80 °C, and 100 °C) for 12 h. After removing the mold, it was cured in an environment with a temperature of 20 ± 2 °C and relative humidity of more than 95% for 7 d. The 7 d compressive strength was determined using a CMT-5105 universal testing machine manufactured by Shandong Luda Testing Instruments Co. (Taian, China), and the results are shown in Table 3.

### 2.4. The Jander Model

#### 2.4.1. Degree of Response

**Figure 4:**
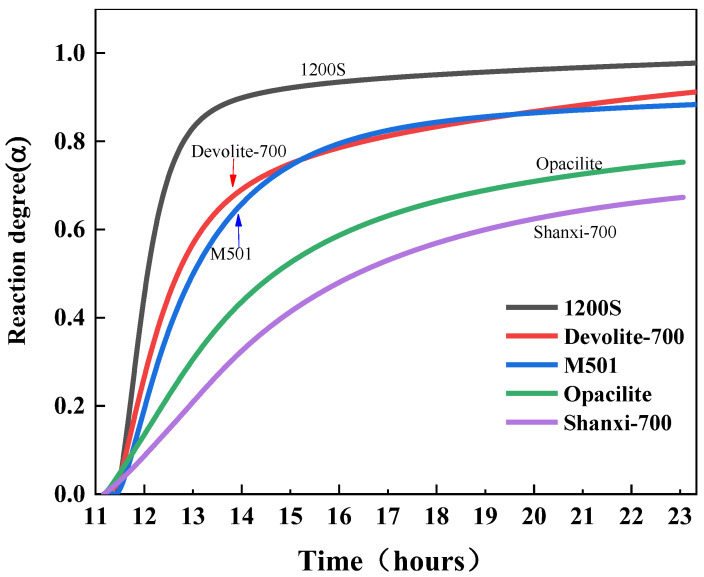
Degree of geological polymerization of metakaolin.

The degree of hydration reaction (Equation (1)) is calculated from the heat of hydration data [21], where *α* is the degree of hydration reaction and *Q_t_* is the cumulative heat release at time *t*. *Q_max_* is the maximum heat release after the hydration reaction (the value of *Q_max_* is determined by fitting Knudsen’s extrapolated Equation (2)). The variation in the degree of reaction *α* with time is shown in Figure 4.

(1) $$\alpha_{t}=\frac{Q_{t}}{Q_{max}}\;$$

(2) $$\frac{1}{Q}=\frac{1}{Q_{max}}+\frac{t_{50}}{Q_{max}\left(t-t_{0}\right)}$$

#### 2.4.2. Reaction Mechanism

Simulation for continuous, possibly overlapping, geopolymerization processes. The rate equation published by Jander was referenced, and the Jander equation was later modified by Kondo by introducing a reaction class *N* to obtain a more widely used hydrodynamic equation [21] (Equation (3)).

(3) $${\left[{1-\left(1-\alpha \right)}^{\frac{1}{3}}\right]}^{N}=K_{N}\cdot t$$

(4) $$lnt=Nln\left[{1-\left(1-\alpha \right)}^{\frac{1}{3}}\right]-lnK_{N}$$

where *K_N_* is the hydration reaction rate constant, *t* is the age of the hydration reaction, and *N* is the regular associated with the hydration mechanism (reaction grade). The reaction control mechanism was formulated based on the *N* value [22]: for *N* < 1, the reaction is controlled by the nucleation–growth mechanism; for *N* = 1, the reaction is controlled by the boundary reaction; and for *N* ≥ 2, the reaction is controlled by the diffusion mechanism.

We take the logarithms of both sides of Equation (3) and readjust it to Equation (4). We plot lnt against $\left[{1-\left(1-\alpha \right)}^{1/3}\right]$ as a linearly fitted line accordingly.

## 3. Results and Discussion

### 3.1. Mechanical Properties

#### 3.1.1. Proportioning Factors Influence the Mechanical Properties of Geopolymers

**Figure 5:**
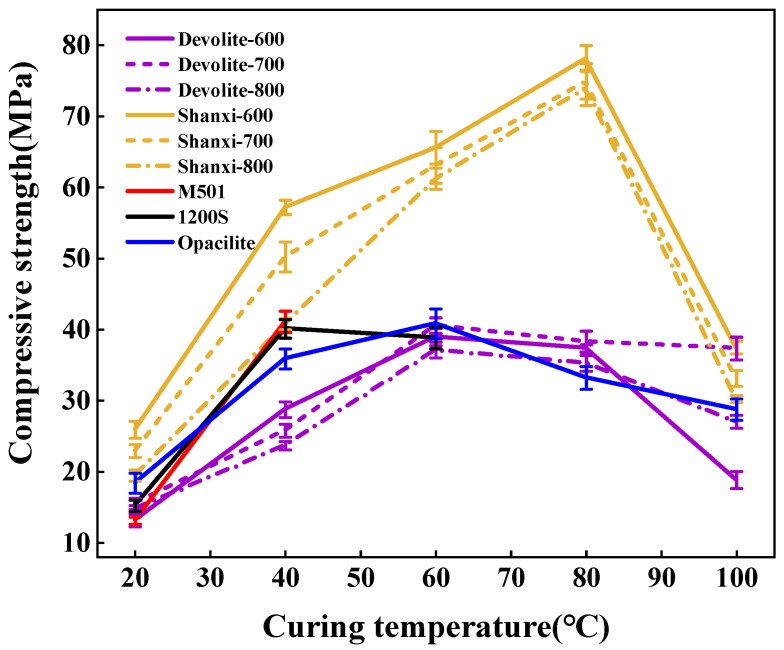
Effect of curing temperature on the compressive strength of geopolymers.

**Figure 6:**
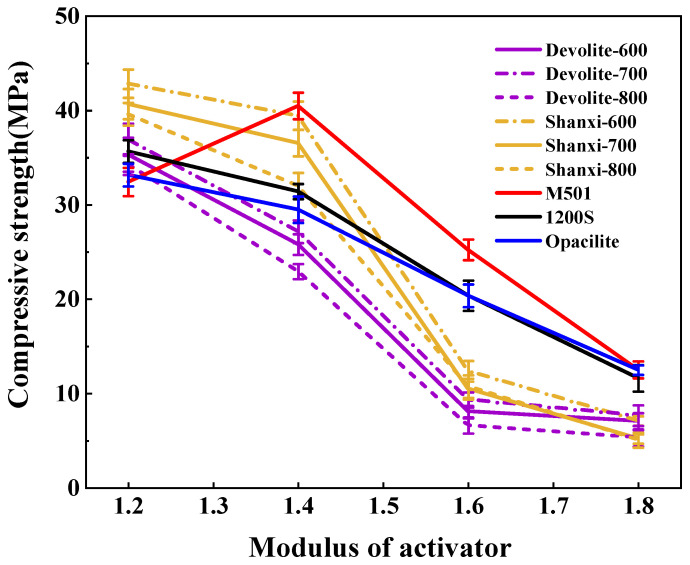
Effect of activator modulus on the strength of geopolymers.

**Figure 7:**
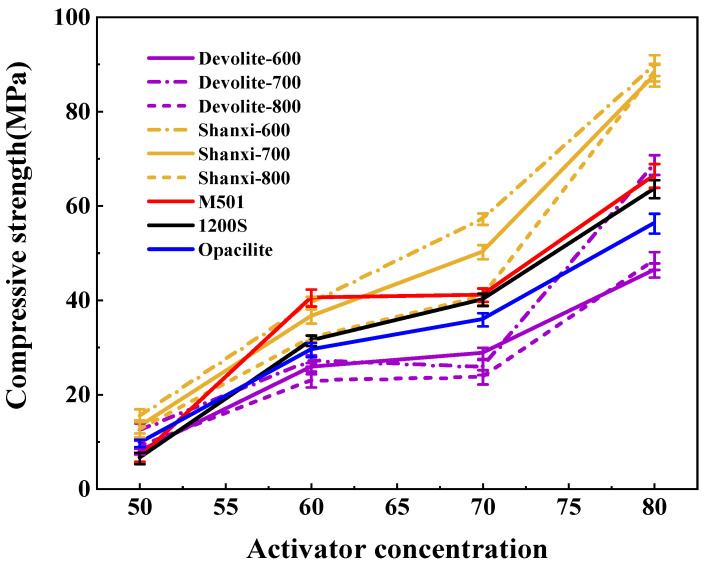
Effect of activator concentration on the strength of geopolymers.

We measured the compressive strength of synthetic geopolymers to investigate the effect of proportioning factors on the mechanical properties of geopolymers. Figure 5, Figure 6 and Figure 7 show the compressive strength of a range of metakaolin-synthesized geopolymers under different external conditions (curing temperature, activator modulus, and activator concentration). Figure 5 shows that as the curing temperature increases, the strength of the geopolymer increases and then decreases. During the initial phase of the geopolymerization reaction, the lower curing temperature does not allow for sufficient activation energy to drive the reaction. The continued increase in curing temperature leads to the capillary collapse of the geopolymer and excessive water loss from the matrix, with more pores and more microcracks, even at 100 °C, when the boiling point of water has been reached. Water dissipation is accelerated, so the strength of the geopolymer decreases. The curing temperature has also been considered one of the main factors affecting the mechanical properties of geopolymers in previous studies [23]. Excessive curing temperatures can lead to the significant loss of moisture from the geopolymer matrix, which forms relatively wide cracks after the deterioration of the geopolymer matrix [24,25]. Figure 6 shows the effect of the activator modulus on the strength of the geopolymer, which decreases as the activator modulus increases. When the modulus is 1.2, the strength of the geopolymer reaches a maximum of 42.86 Mpa. At this time, the activator is more alkaline. The number of highly polymerized siloxo-tetrahedral structural groups in the system decreases. In contrast, the number of oligomeric siloxo-tetrahedral structural groups that facilitate the occurrence of solid bonding reactions gradually increases; specifically, the presence of Q^0^ island siloxo-tetrahedral oligomeric structures has an obvious promotion effect on the geopolymerization reaction. A better bonding reaction corresponds to the higher strength of the geopolymer [26]. However, at a modulus of 1.8, the strength of the geopolymer is substantially reduced due to the high content of sodium silicate solution in the high-modulus activator, causing the geopolymer slurry to become dense and weakly alkaline, which hinders the depolymerization and polymerization of the gelling material and inhibits the production of N-A-S-H. Figure 7 depicts the effect of the activator concentration on the strength of the geopolymer. At an 80% alkali activator concentration, the system leached a large amount of Al^3+^, Si^4+^, and other trace ions, which led to the formation of the Si(OH)_4_ monomer and [Al(OH)_4_]^−^ monomer, and the strength of the geopolymer reached 89.74 MPa. When the alkali activator concentration is high, the leaching of Al^3+^, Si^4+^, and other trace ions increases, forming more geopolymer gels and thus increasing the strength of the geopolymers [27]. However, low alkali activator concentrations are insufficient to break the Si-Al bonds in the silica–aluminate, and only small amounts of aluminates and silicate tetrahedral monomers are produced [28]. Therefore, the system had a weak chemical reaction when the alkali activator concentration was 50%, leading to lower geopolymer strength.

#### 3.1.2. Physicochemical Properties Affect the Mechanical Properties of Geopolymers

**Figure 8:**
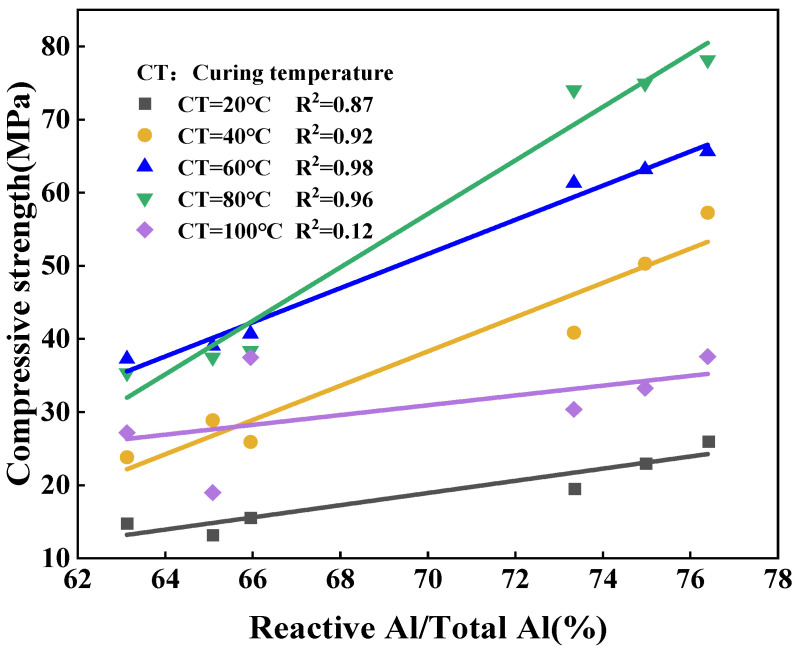
Correlation of ground polymer strength with active/total aluminum content in metakaolin.

As shown in Figure 5, Figure 6 and Figure 7, the strength of the synthesized geopolymers differs due to differences in the physicochemical properties of the series of biased kaolins at the same curing temperature, activator modulus, and activator concentration. On the one hand, as shown in Table 2 and Table 3, the amorphous content of Shanxi-700 and M501 was 89.73 and 89.27, but the strength of the two synthetic geopolymers differed considerably, even at a curing temperature of 60–100 °C, where the latter had almost no strength due to the development of significant cracks. Due to the different chemical and mineralogical properties of kaolin with different particle sizes and specific surface areas, the smaller the particle size and the larger the specific surface area, the easier it is for the alkali activator to come into contact with it, thus releasing more silica–aluminum monomers. On the other hand, the Devolite and Shanxi metakaolin materials were calcined at high temperatures from 600 to 800 °C, when the content of six-coordinated Al atoms decreased. In contrast, the content of structurally unstable four-coordinated Al atoms gradually increased [29]. Therefore, different calcination temperature conditions lead to differences in the number of four-, five-, and six-coordinated Al atoms in the metakaolin, which results in various strengths of the synthetic geopolymers from Devolite- and Shanxi-type metakaolin materials. As can be seen from Figure 8, there is a significant correlation between the power of the ground polymer prepared from the same kaolin clay after calcination at different temperatures and their reactive Al content (reactive Al also acts as an essential component of the amorphous form of metakaolin; the 100 °C correlation of 0.12 is probably due to an experimental error). The test results show that the Si/Al (active) molar ratio is another critical factor influencing the development of the strength of the geopolymer. Table 2 and Table 3 show that the Devolite-700 geopolymer, with a smaller Si/Al (active) molar ratio, has higher power when the internal and external conditions of the Devolite-700 and Devolite-800 hydration processes are the same. Because, in geopolymer systems with relatively low Si/Al (active) moles, the [Al(OH)_4_]^−^ monomer does not appear during the initial phase of the reaction, the [Al(OH)_4_]^−^ monomer and [SiO(OH)_3_]^−^ monomer are rapidly condensed to form silicoaluminate oligomers. A large amount of oligomer condensation allows the silicoaluminate network to be quickly established.

### 3.2. Microstructure

#### 3.2.1. FTIR

**Figure 9:**
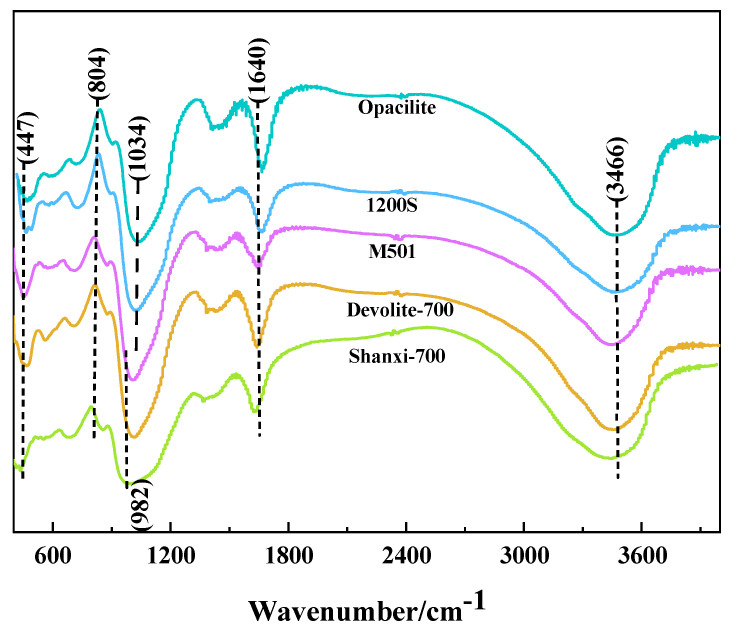
FTIR of different metakaolin-based geopolymers.

Figure 9 shows that, comparing the FTIR spectra of the five metakaolin-based polymers, all samples produced Si-O-T (T = Si or Al) asymmetric stretching vibrations at 950–1200 cm^−1^. Within this range, the vibration peak of Shanxi-700 shifts from 1034 cm^−1^ to 982 cm^−1^ with the largest offset. This implies an increase in the Al replacing Si in the structure of the Shanxi-700 reactants, which distorts the vibrations [30]. The Si-O-T (T = Si or Al) asymmetric stretching vibration at this point shifts in the direction of lower frequencies, which indicates the occurrence of a geopolymerization reaction with the formation of a silica–aluminate gel [5]. The vibrational peak at 804 cm^−1^ is attributed to the symmetric stretching vibration of Si-O-Si in quartz; at 447 cm^−1^, it corresponds to the bending vibration of Si-O-Si in Si-rich glasses or quartz [31]. A longitudinal comparison of the characteristic peaks at 447 cm^−1^ and 804 cm^−1^ shows that the peak intensity of Shanxi-700 is weaker. In contrast, the Opacilite characteristic peak is stronger, probably due to the smaller Si/Al of the former and the larger Si/Al of the latter. The results of the strength tests also prove that the Shanxi-700 geopolymer is stronger. The water molecule absorption band at 3466 cm^−1^ is associated with the presence of structural and free water in the matrix and corresponds to the stretching vibration of [OH]^−^; the water molecule absorption band at 1640 cm^−1^ corresponds to the bending vibration of H-O-H [5,29]. The vibrational peaks of these water molecules reflect the uptake of water in the matrix, with the highest intensity at 1640 cm^−1^ for the Opacilite geopolymer, indicating that the matrix is more water-rich and, therefore, the structure of the Opacilite matrix is not denser.

#### 3.2.2. SEM

**Figure 10:**
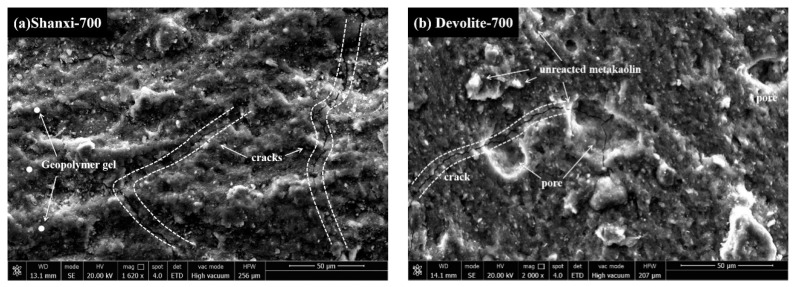

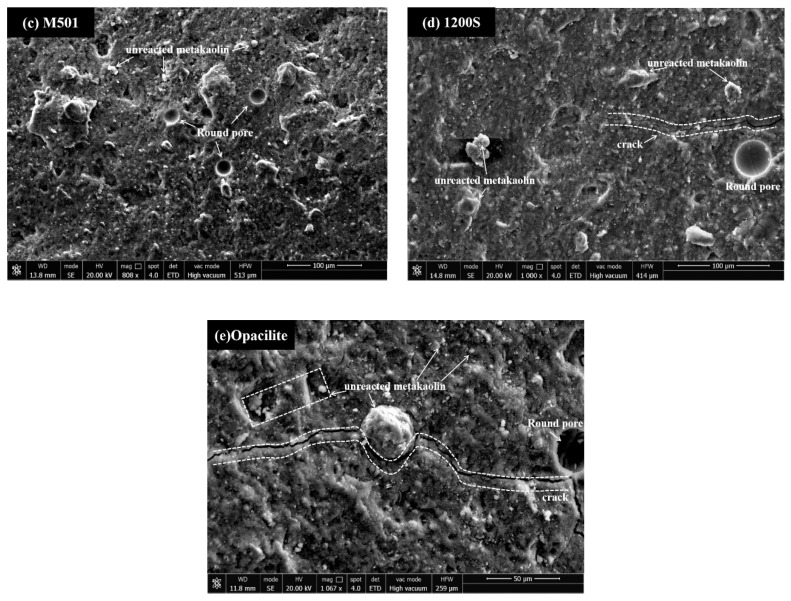
SEM images of geopolymers synthesized with different MK at M = 1.4, C = 60% conditions.

This study selected samples of different metakaolin clays at the same preparation conditions (modulus of 1.4 and concentration of 60%) to estimate the degree of hydration of different metakaolin synthetic geopolymers by SEM. As shown in Figure 10, almost all the substrates show microcracks or pores, which are due to the formation of pore channels caused by the evaporation of free water from the substrate. Figure 10b,d show distinct crater features, which are expected due to the formation of incompletely expelled air bubbles as a result of vibratory pounding. The better the homogeneity of the substrate, the higher the corresponding degree of hydration and, in macro terms, the higher the compressive strength. Matrix **e** in the diagram has the most apparent cracks and corresponds to lower strength. In contrast, in Figure 10c, no noticeable pores or microcracks are observed, and gel agglomeration makes the matrix more dense, thus reaching a maximum power of 40.49 Mpa. Microcracks can be seen in Figure 10a, but the overall lamellar structure is disintegrated and replaced by a large and dense gel phase; therefore, a higher degree of hydration is achieved. Incompletely reacted metakaolin particles can be observed adhering to the surface of the matrix in Figure 10b,d, indicating that the dissolution is not complete in the pre-hydration phase. Meanwhile, the cross-section of the matrix in Figure 10d shows better flatness and a denser structure compared to Figure 10b.

### 3.3. Heat of Hydration

#### 3.3.1. Rate of Exothermic Hydration

**Figure 11:**
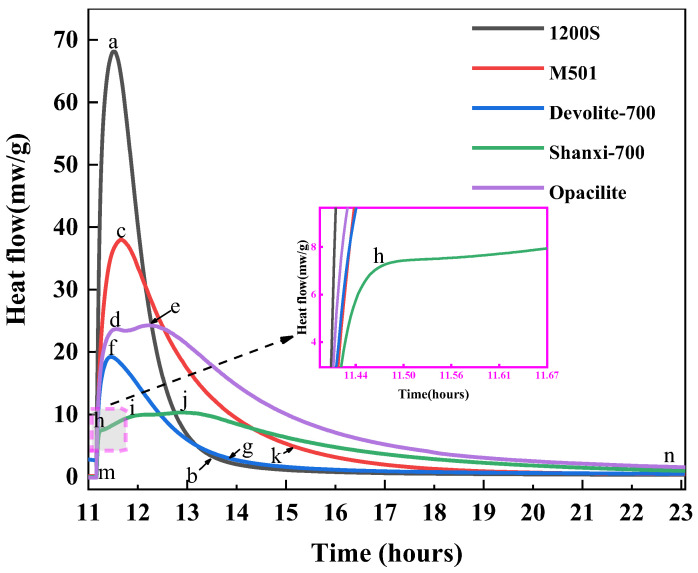
Hydration heat release rates of different metakaolin materials.

As shown in Figure 11, only one exothermic peak occurs in the pre-exothermic hydration phase of the 1200S, M501, and Devolite-700 types of metakaolin. The surface and interior of the metakaolin particles at this point absorb the water component of the liquid phase, resulting in the wetting and dissolution of the metakaolin [32]. It has also been suggested that the early dissolution exothermic peak is usually a physical rather than a chemical reaction process [33]. There is only one exothermic peak in the hydration exothermic process, which can be attributed to the rapid development of the geological polymerization process, resulting in the overlapping of the reaction steps, with the next accelerated “polymerization” stage taking place very quickly. The 1200S material has a smaller particle size and larger specific surface area, so it has the most considerable exothermic rate. However, at the later stage of hydration, the exothermic rate of hydration of 1200S was significantly smaller than that of M501 and Devolite-700. This is due to the speedy dissolution and condensation process of 1200S, leading to the generation of N-A-S-H encapsulated on the surfaces of the metakaolin particles. This hinders the erosive effect of the hydroxyl groups on the metakaolin particles in the solution [34].

The first exothermic peak of the Shanxi-700 metakaolin is closely followed by the second exothermic peak, which indicates that the ionic concentration has reached a saturated state within a short period and, therefore, the induction period of hydration is not significant. It can be concluded that the appearance of the induction period in geopolymer hydration is mainly attributed to changes in the solubility of the system only when the system transitions from an undersaturated to a saturated or oversaturated state, and the products of the geopolymerization process are gradually precipitated. As the concentration of ions in the system continues to increase, the rate of dissolution decreases again, resulting in slower rates of dissolution at later stages, and an induction period is thus formed.

The hydration of Opacilite was observed as a dissolution peak, a central exothermic peak, and another exothermic peak. In the pre-hydration stage, the exothermic rate of hydration of Opacilite is lower than that of 1200S and M501, which is correlated with Si/Al. It has been shown [35,36] that in the range of 1.15 < Si/Al < 1.90, the density of the polymer increases, and the mechanical strength grows linearly and it is more fully reacted. However, M501 has less Si/Al than Opacilite, yet it has a stronger hydration reaction capability. This is because the contact area within M501 has a smaller average particle size, and the liquid is relatively more significant for the same mass, which promotes faster heat release. In the later stages of hydration, the geopolymer gel structure was further reorganized locally, resulting in a more structurally stable silica–aluminate gel phase, which could be one of the reasons for the formation of the third exothermic peak.

#### 3.3.2. Accumulated Heat Release from Hydration

**Figure 12:**
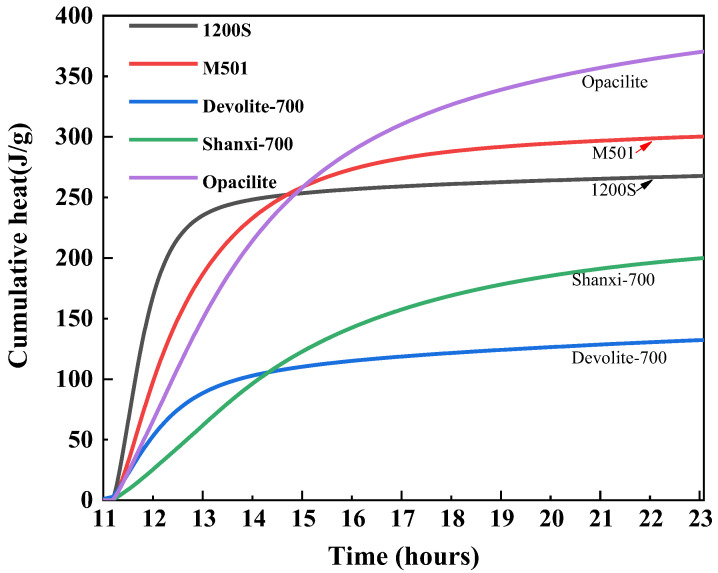
Cumulative heat release of different metakaolin materials.

In Figure 12, the relatively gentle exothermic rate of Shanxi-700 results in higher total cumulative heat release and, therefore, the highest strength of the synthetic geopolymers. The 1200S material has the most rapid ‘dissolution’ phase, which is reflected in the initial rise in cumulative heat, after which the total heat of the reaction does not change. After 15.42 h, when the total exotherm of Shanxi-700 exceeds 1200S, the differences in the various exotherms become more pronounced. Due to the accelerated exotherm during the ‘polymerization’ process, the total cumulative exotherm of Opacilite after 14.6 h is greater than the total exotherm of Devolite-700. Therefore, the Devolite-700 synthetic geopolymer is the strongest.

### 3.4. Hydration Kinetics

#### 3.4.1. Number of Reaction Levels N

**Figure 13:**
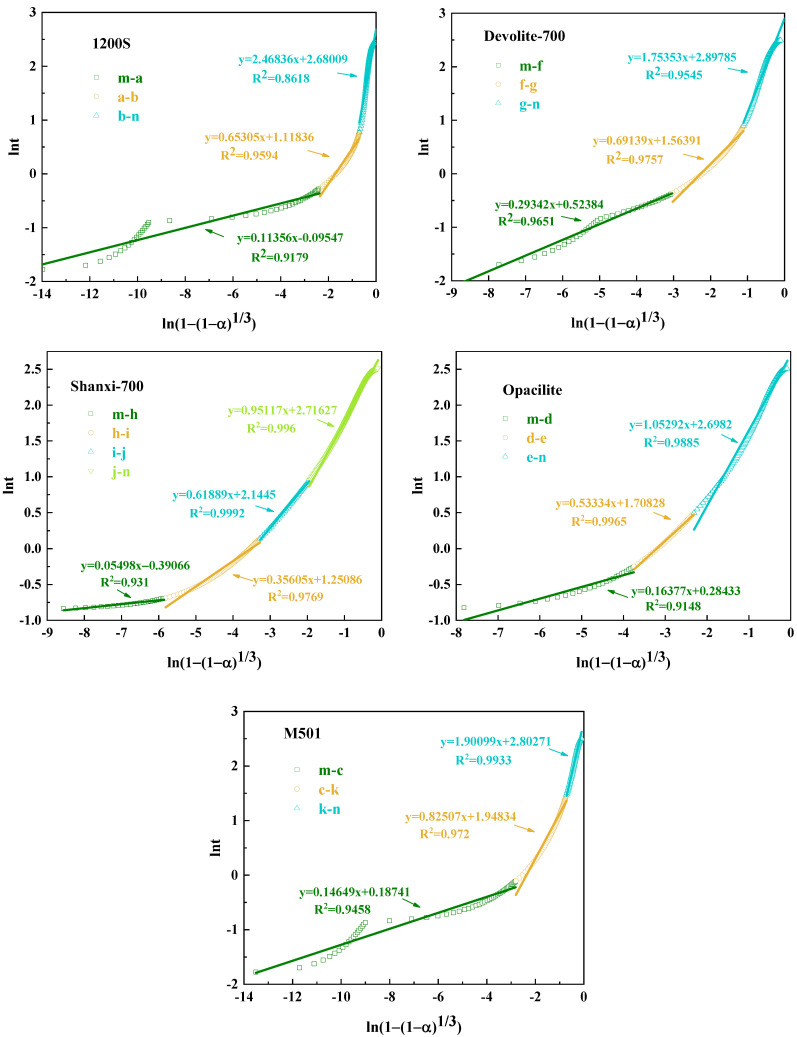
Jander simulation of geological polymerization of different metakaolin types.

As can be seen from Figure 13 and Table 4, during the hydration exothermic reaction, 1200S (m-a, a-b segments), Devolite-700 (m-f, f-g segments), M501 (m-c, c-k segments), Shanxi-700 (m-d, d-e segments), and Opacilite (m-h, h-i, i-j, j-n segments) have *N* < 1. At this time, the hydration exothermic process is dominated by the control of the nucleation–growth mechanism, which is essential in the preliminary stage of the geopolymerization reaction. The nuclei of the hydration reaction products are first generated randomly at the solid–liquid interface, and the nuclei with isotropy grow gradually at the constant interface. Afterward, the exothermic process of hydration changes from an accelerated reaction phase to a recession phase, where the hydration products continue to grow on top of the original products but do not form new nuclei [37]. From the point of view of nucleation growth, it can be explained that the overlapping of mutual contacts between different nuclei leads to the termination of the nucleation process. Opacilite, which has the lowest degree of hydration reaction, is dominated throughout the hydration process by the nucleation–growth mechanism. This is because the hydration of Opacilite is extremely slow, and the development of the gel layer from thin to thick hinders ionophore motion binding due to the massive encapsulation of the new phase. Even during accelerated condensation at the central exothermic peak of the reaction, a relatively slow reaction rate is maintained.

The *N* values of Devolite-700 (g-n section) and M501 (k-n section) are 1.75 and 1.9, respectively, and the hydration process is stable. At this point, the generated gel phase will cover the biotite particles, and the hydration exothermic reaction is more inclined to be dominated by the diffusion mechanism. This is shared by the descriptive model of the silicoaluminate hydration process proposed in the literature [38]: in the later stages of hydration, diffusion controls the reaction. For 1200S (b-n segment), *N* > 2, and the reaction process is controlled by diffusion. The diffusion of the liquid phase through the hydration product layer is one of the main factors controlling the reaction [19]. The denser the hydration product layer, the more excellent the diffusion resistance and the higher the *N* value. The exothermic process of hydration of 1200S gradually transitions to the stable phase, probably because the hydration product layer accumulated by 1200S in the accelerated phase is too thick. Its permeability is minimal, which makes it difficult for the liquid phase to penetrate through the product layer, and, thus, the *N* value reaches a maximum of 2.47.

#### 3.4.2. The Reaction Rate Constant K_N_

The hydration rate constant *K_N_* is an essential parameter in chemical kinetics; the magnitude of its value directly reflects the speed of the rate and can reflect the rate characteristics of the reaction system. Table 4 shows that the *K_N_* values during the initial hydration period are in descending order of Opacilite > 1200S > M501 > Shanxi-700 > Devolite-700, which, in conjunction with Table 2, indicates that the larger the specific surface area and the smaller the particle size, the more likely it is that the partial kaolin will react early in the ground polymerization. On the other hand, the *K_N_* values during the initial phase of hydration are much greater than those during the recession or stabilization phase of the hydration reaction, which also implies that the rate of nucleation–growth in the early stages of geosynthesis far exceeds the diffusion rate.

In addition, the *K_N_* values are usually influenced by the temperature. The reaction process is subject to a combination of the external ambient temperature and the reaction exotherm, resulting in a relatively higher reaction rate for the relatively high exotherm of metakaolin [39]. However, Figure 12 shows that Shanxi-700 has the most significant heat release but does not have the largest *K_N_* value. This is because the influencing factor controlling the magnitude of the *K_N_* value is not only the reaction temperature; it is also related to the nature of the reactants and the reaction medium. During the recession phase, the system heats up due to the gradual accumulation of heat released from hydration, while the *K_N_* value decreases. This is probably due to the increase in hydration products, the progressive control of the reaction by diffusion, and the increased diffusion resistance due to the dense structure, resulting in a lower *K_N_* value.

## 4. Conclusions

The focus of this study was the evaluation of the mechanical properties and reaction kinetics of synthetic ground polymers of metakaolin. The geopolymerization process was monitored by isothermal calorimetry, and the issue of reaction control mechanisms was investigated using the Jander model. The following conclusions were obtained from the experimental results.

(1) The compressive strength of the geopolymer is influenced by a combination of the proportioning factors and the physical and chemical properties of the raw material. We used different calcination conditions for the same metakaolin, thus establishing a correlation between the physicochemical properties of the metakaolin and the strength of the geopolymer, where the correlation between the amorphous content and the compressive strength of the geopolymer exceeds 0.87.
(2) We obtained a segmented fit of the exothermic rate curve for metakaolin, with increasing *N* and decreasing *K_N_* values of the reaction rate constant, for the synthesis of the geopolymer. The early nucleation–growth rate of geological aggregation far exceeds the later diffusion rate
(3) In the early stages of the synthesis of metakaolin geopolymers, the geopolymerization reactions are controlled by the nucleation–growth mechanism, favoring the growth of nuclei in the hydration products and progressively accelerating the rate of hydration. In the later stages of the hydration reaction, the boundary reaction or diffusion mechanism dominates the reaction, and the larger *N* values in the latter stages of the metakaolin reaction are due to the formation of a denser layer of hydration products and greater diffusion resistance.
(4) The *K_N_* values of the reaction rate constants during the initial hydration period demonstrate that the larger the specific surface area and the smaller the particle size, the more likely it is that the kaolin will “dissolve” early in the ground polymerization process. Diffusion in the later stages of the hydration reaction leads to a reduction in the reaction rate constant *K_N_*.

## Figures and Tables

**Table 1:** Chemical composition of kaolin and metakaolin (wt%).

Varieties	Al_2_O_3_	SiO_2_	Fe_2_O_3_	MgO	K_2_O	Na_2_O	Others
Devolite	37.73	50.73	1.12	0.27	1.65	0.24	8.26
Shanxi	41.12	50.34	0.59	0.58	0.14	0.13	7.10
M501	42.35	49.68	0.50	0.01	0.16	0.19	7.11
1200S	39.16	55.13	1.80	0.15	0.56	0.65	2.55
Opacilite	40.92	51.57	0.52	0.24	1.38	0.27	5.10

**Table 2:** Physicochemical properties of different MK.

Varieties	Amorphous Phase Content (%)	Reactive Al/ Total Al (%)	D (um)	Si/Reactive Al	LOI (g)	BET (m^2^/g)	Q (%)
Devolite-600	82.14	65.09	54.20	1.74	1.32	6.58	1.74
Shanxi-600	91.59	76.42	44.40	1.36	2.12	13.99	2.32
Devolite-700	88.13	65.96	54.20	1.73	1.58	6.58	1.74
Shanxi-700	89.73	74.99	44.40	1.38	2.07	13.99	2.32
Shanxi-800	88.62	73.36	44.40	1.40	1.93	13.99	2.32
Devolite-800	87.79	63.13	54.20	1.77	1.65	6.58	1.74
M501	89.27	88.62	29.00	1.13	2.54	13.90	0.76
1200S	84.67	80.21	21.70	1.50	1.53	19.00	0.53
Opacilite	69.74	56.79	52.90	1.89	2.32	10.98	1.62

**Table 3:** Compressive strength of geopolymer synthesized with different metakaolin materials under mixture ratio (MPa).

External Influencing Factors	Curing Temperature (°C) M = 1.4 C = 70%					Modulus of Activator T = 40 °C C = 60%				Concentration of Activator (%) T = 40 °C M = 1.4	
Varieties	Ambient	40	60	80	100	1.2	1.4	1.6	1.8	50	80
Devolite-600	13.14	28.75	38.94	37.36	18.84	35.32	25.80	8.07	7.03	7.92	46.33
Shanxi-600	25.92	57.20	65.58	78.14	37.47	42.86	39.46	12.36	7.09	15.40	89.74
Devolite-700	15.51	25.77	40.56	38.28	37.34	36.91	27.16	9.32	7.66	12.36	68.67
Shanxi-700	22.92	50.21	63.09	74.92	33.13	40.69	36.55	10.47	5.18	13.21	87.59
Devolite-800	14.73	23.67	37.12	35.25	27.03	34.28	22.93	6.56	5.29	8.93	48.33
Shanxi-800	19.47	40.77	61.23	74.01	30.23	39.60	32.01	10.73	5.06	12.56	88.25
M501	13.12	41.09	-	-	-	32.43	40.49	25.23	12.51	6.70	66.37
1200S	15.21	40.11	38.80	-	-	35.65	31.41	20.37	11.62	6.50	63.55
Opacilite	18.40	35.88	40.81	33.20	28.75	33.16	29.49	20.36	12.49	9.71	56.25

Note: “-” indicates that the reaction product is of very low strength due to the presence of visible cracks. As shown in Figure 3, the QUANTA 650 FEG field emission ambient scanning electron microscope from FEI, Hillsboro, OR, USA, was used to observe the geopolymer morphology state and infrared qualitative analyses were carried out using the Nicolet IS10 infrared spectrometer from Thermo Fisher, Waltham, MA, USA. An eight-channel isothermal microcalorimeter 3114/3236 TAM Air Sweden from Thermometric AB, Stockholm, Sweden, was used to measure the exothermic behaviour of the reaction of five metakaolin clays, Devolite-700, Shanxi-700, M501, 1200S and Opacilite, at a 40 °C maintenance temperature. The effect of the nature of the feedstock components on the geological polymerization reaction was analysed using exothermic curves. ICC recorded the exothermic rates for reaction times up to 23 h.

**Table 4:** Hydration kinetics parameters of geopolymers.

Specimen	Subsection	Slope (N)	Intercept (−lnk)	k	R^2^
1200S	m-a	0.11356	−0.09547	1.10017	0.91827
	a-b	0.65305	1.11836	0.32681	0.95952
	b-n	2.46836	2.68009	0.06855	0.92836
Devolite-700	m-f	0.29342	0.52384	0.59224	0.96524
	f-g	0.69139	1.56391	0.20931	0.97574
	g-n	1.75353	2.89785	0.05514	0.9545
M501	m-c	0.14649	0.18741	0.82910	0.94601
	c-k	0.82507	1.94834	0.14251	0.97201
	k-n	1.90099	2.80271	0.06064	0.99333
Shanxi-700	m-d	0.16377	0.28433	0.75251	0.91552
	d-e	0.53334	1.70828	0.18117	0.9965
	e-n	1.05292	2.6982	0.06732	0.98855
Opacilite	m-h	0.05498	−0.39066	1.47795	0.93392
	h-i	0.35605	1.25086	0.28625	0.97696
	i-j	0.61889	2.1445	0.11712	0.99917
	j-n	0.95117	2.71627	0.06612	0.99596

## Data Availability

Data are contained within the article.

## References

[1] Xie J., Wu Z., Zhang X., Hu X., Shi C. (2023). Trends and Developments in Low-Heat Portland Cement and Concrete: A Review. Constr. Build. Mater..

[2] Xue L., Zhang Z., Walkley B., Liu H., Jiang Y., Wang H. (2023). Retarding Effect of Gypsum for Hybrid Alkali-Activated Cements (HAACs) at Ambient Temperature. Mater. Today Commun..

[3] Ahmed H.U., Mohammed A.S., Qaidi S.M.A., Faraj R.H., Hamah Sor N., Mohammed A.A. (2023). Compressive Strength of Geopolymer Concrete Composites: A Systematic Comprehensive Review, Analysis and Modeling. Eur. J. Environ. Civ. Eng..

[4] Paruthi S., Husain A., Alam P., Husain Khan A., Abul Hasan M., Magbool H.M. (2022). A Review on Material Mix Proportion and Strength Influence Parameters of Geopolymer Concrete: Application of ANN Model for GPC Strength Prediction. Constr. Build. Mater..

[5] Dai S., Wang H., An S., Yuan L. (2022). Mechanical Properties and Microstructural Characterization of Metakaolin Geopolymers Based on Orthogonal Tests. Materials.

[6] Xue L., Zhang Z., Wang H. (2021). Early Hydration Kinetics and Microstructure Development of Hybrid Alkali Activated Cements (HAACs) at Room Temperature. Cem. Concr. Compos..

[7] Cao R., Zhang S., Banthia N., Zhang Y., Zhang Z. (2020). Interpreting the Early-Age Reaction Process of Alkali-Activated Slag by Using Combined Embedded Ultrasonic Measurement, Thermal Analysis, XRD, FTIR and SEM. Compos. Part B Eng..

[8] Hu Z., Wyrzykowski M., Lura P. (2020). Estimation of Reaction Kinetics of Geopolymers at Early Ages. Cem. Concr. Res..

[9] Glosser D., Suraneni P., Isgor O.B., Weiss W.J. (2020). Estimating Reaction Kinetics of Cementitious Pastes Containing Fly Ash. Cem. Concr. Compos..

[10] Kurtulus C., Baspinar M.S. (2023). An Essential Study of Strength Development in Geopolymer Materials Using the JMAK Method. Arab. J. Sci. Eng..

[11] Krstulović R., Dabić P. (2000). A Conceptual Model of the Cement Hydration Process. Cem. Concr. Res..

[12] HAN F., WANG D., YAN P. (2014). Hydration Kinetics of Composite Binder Containing Different Content of Slag or Fly Ash. J. Chin. Ceram. Soc..

[13] Provis J.L. (2016). On the Use of the Jander Equation in Cement Hydration Modelling. RILEM Tech. Lett..

[14] Sun Z., Vollpracht A. (2018). Isothermal Calorimetry and In-Situ XRD Study of the NaOH Activated Fly Ash, Metakaolin and Slag. Cem. Concr. Res..

[15] Nath S.K., Kumar S. (2017). Reaction Kinetics, Microstructure and Strength Behavior of Alkali Activated Silico-Manganese (SiMn) Slag—Fly Ash Blends. Constr. Build. Mater..

[16] Ravikumar D., Neithalath N. (2012). Reaction Kinetics in Sodium Silicate Powder and Liquid Activated Slag Binders Evaluated Using Isothermal Calorimetry. Thermochim. Acta.

[17] Nath S.K., Mukherjee S., Maitra S., Kumar S. (2017). Kinetics Study of Geopolymerization of Fly Ash Using Isothermal Conduction Calorimetry. J. Therm. Anal. Calorim..

[18] Nath S.K., Kumar S. (2019). Role of Alkali Concentration on Reaction Kinetics of Fly Ash Geopolymerization. J. Non-Cryst. Solids.

[19] Zhang C., Hu Z., Wang X., Zhu H., Yang X., Gu X. (2020). Hydration Heat and Hydration Kinetics of Silane Coupling Agent/Metakaolin Based Geopolymers. Mater. Rep..

[20] Peng H., Cui C., Cai C., Li S., Zhang X. (2014). Research on Influence of Calcination Temperature on Metakaolin Reactivity and Its Determination. Bull. Chin. Ceram. Soc..

[21] Chen C., Gong W., Lutze W., Pegg I.L., Zhai J. (2011). Kinetics of Fly Ash Leaching in Strongly Alkaline Solutions. J. Mater. Sci..

[22] Fernández-Jiménez A., Puertas F. (1997). Alkali-Activated Slag Cements: Kinetic Studies. Cem. Concr. Res..

[23] Sajan P., Jiang T., Lau C., Tan G., Ng K. (2021). Combined Effect of Curing Temperature, Curing Period and Alkaline Concentration on the Mechanical Properties of Fly Ash-Based Geopolymer. Clean. Mater..

[24] Atiş C.D., Görür E.B., Karahan O., Bilim C., İlkentapar S., Luga E. (2015). Very High Strength (120MPa) Class F Fly Ash Geopolymer Mortar Activated at Different NaOH Amount, Heat Curing Temperature and Heat Curing Duration. Constr. Build. Mater..

[25] Yomthong K., Wattanasiriwech D., Aungkavattana P., Wattanasiriwech S. (2021). Effect of NaOH Concentration and Curing Regimes on Compressive Strength of Fly Ash-Based Geopolymer. Mater. Today Proc..

[26] Cao D., Su D., Song G. (2004). Geopolymeric Behavior and Structure of Lower Modulus Sodium Silicate Solutions. Kuei Suan Jen Hsueh Pao/J. Chin. Ceram. Soc..

[27] Abdullah S.F.A., Yun-Ming L., Bakri M.M.A., Cheng-Yong H., Zulkifly K., Hussin K. (2018). Effect of Alkali Concentration on Fly Ash Geopolymers. IOP Conf. Ser. Mater. Sci. Eng..

[28] Abdullah A., Hussin K., Abdullah M.M.A.B., Yahya Z., Sochacki W., Razak R.A., Błoch K., Fansuri H. (2021). The Effects of Various Concentrations of NaOH on the Inter-Particle Gelation of a Fly Ash Geopolymer Aggregate. Materials.

[29] Longhi M.A., Walkley B., Rodríguez E.D., Kirchheim A.P., Zhang Z., Wang H. (2019). New Selective Dissolution Process to Quantify Reaction Extent and Product Stability in Metakaolin-Based Geopolymers. Compos. Part B Eng..

[30] Prasanphan S., Wannagon A., Kobayashi T., Jiemsirilers S. (2019). Reaction Mechanisms of Calcined Kaolin Processing Waste-Based Geopolymers in the Presence of Low Alkali Activator Solution. Constr. Build. Mater..

[31] Zhang Z., Wang H., Provis J.L. (2012). Quantitative Study of the Reactivity of Fly Ash in Geopolymerization by FTIR. J. Sustain. Cem. -Based Mater..

[32] Duxson P., Fernández-Jiménez A., Provis J.L., Lukey G.C., Palomo A., van Deventer J.S.J. (2007). Geopolymer Technology: The Current State of the Art. J. Mater. Sci..

[33] Gao X., Yu Q.L., Brouwers H.J.H. (2015). Reaction Kinetics, Gel Character and Strength of Ambient Temperature Cured Alkali Activated Slag–Fly Ash Blends. Constr. Build. Mater..

[34] Chen X., Mondal P. (2020). Effects of NaOH Amount on Condensation Mechanism to Form Aluminosilicate, Case Study of Geopolymer Gel Synthesized via Sol–Gel Method. J. Sol.-Gel Sci. Technol..

[35] Duxson P., Provis J.L., Lukey G.C., Mallicoat S.W., Kriven W.M., van Deventer J.S.J. (2005). Understanding the Relationship between Geopolymer Composition, Microstructure and Mechanical Properties. Colloids Surf. A Physicochem. Eng. Asp..

[36] Zhang Z., Wang H., Provis J.L., Bullen F., Reid A., Zhu Y. (2012). Quantitative Kinetic and Structural Analysis of Geopolymers. Part 1. The Activation of Metakaolin with Sodium Hydroxide. Thermochim. Acta.

[37] Zhang Z. (2020). Study on the Hydration Kinetics Model of Cement-Slag Composite Cementitious Materials. Ph.D. Thesis.

[38] Fernández-Jiménez A., Palomo A., Criado M. (2005). Microstructure Development of Alkali-Activated Fly Ash Cement: A Descriptive Model. Cem. Concr. Res..

[39] Wang X., Zhang C., Zhu H., Wu Q. (2022). Reaction Kinetics and Mechanical Properties of a Mineral-Micropowder/Metakaolin-Based Geopolymer. Ceram. Int..

